# Residue-Specific Dock-Loosen-Unfold Mechanism of GB1 on Nanoparticle Surfaces Revealed by Kinetic and Φ-Value Analysis

**DOI:** 10.3390/biom16010114

**Published:** 2026-01-08

**Authors:** Tingting Liu, Yunqiang Bian, Siyu Wang, Yang Li, Yi Cao, Yonghua Jiao, Hai Pan

**Affiliations:** 1 Wenzhou Institute, University of Chinese Academy of Sciences, Wenzhou 325001, China; 2301509@stu.neu.edu.cn (T.L.); yqbian@ucas.ac.cn (Y.B.); 2201444@stu.neu.edu.cn (S.W.); liyangchem@foxmail.com (Y.L.); caoyi@nju.edu.cn (Y.C.); 2 College of Life and Health Sciences, Northeastern University, Shenyang 110819, China; 3 Chemistry and Biomedicine Innovation Center, Nanjing University, Nanjing 210093, China

**Keywords:** protein–nanoparticle interactions, surface-induced unfolding, adsorption kinetics, Φ-value mapping

## Abstract

Nanoparticles interact dynamically with proteins, often leading to adsorption-induced conformational changes that alter protein function and contribute to corona formation. Here we investigated the adsorption and unfolding of a model protein GB1 on latex nanoparticle surfaces using a combination of mutational analysis, equilibrium binding assays, stopped-flow kinetics and Φ-value interpretation. Seven site-directed variants of GB1 were studied to dissect residue-specific contributions to adsorption energetics. Fluorescence binding isotherms revealed that D46A and T53A mutations weakened surface affinity, while kinetic analysis demonstrated that D46A reduced adsorption rate by ~6-fold and produced a dramatic unfolding/refolding shift, identifying Asp46 as a key docking site. Φ-value analysis further highlighted Asp46 and Thr53 as central residues in the adsorption transition state, whereas mutations in the hydrophobic core or distal loops had negligible effects. These results support a dock–loosen–unfold mechanism in which electrostatic recognition initiates binding, followed by hydrophobic exposure and hairpin stabilization. This residue-level sampling of key sites advances mechanistic understanding of protein–nanoparticle interactions and suggests strategies for tuning surface charge to control corona formation. Our approach provides a generalizable method to map adsorption transition states, with implications for designing safer nanomaterials, predicting protein corona composition, and harnessing protein unfolding in biosensing applications.

## 1. Introduction

Nanoparticles have emerged as versatile tools in nanomedicine, biosensing, and materials engineering owing to their tunable surface properties and high surface-to-volume ratios [1,2,3,4,5]. However, upon exposure to biological environments, nanoparticles rapidly adsorb proteins to form a corona that governs their biological identity and function [3,6,7]. This adsorption process is often accompanied by protein conformational changes that can reduce protein functionality, promote aggregation, or trigger immune responses [8,9,10,11]. Understanding the molecular basis of protein–nanoparticle interactions is therefore critical for both mitigating nanotoxicity and leveraging nanoparticle–protein complexes in therapeutic and diagnostic applications [12,13,14].

A longstanding challenge lies in deconvoluting the dual contributions of adsorption and conformational change, which often occur on overlapping timescales [15,16,17,18,19]. Thermodynamic measurements provide important information about binding equilibria, yet they may fail to capture transient intermediates or rapid structural transitions that define the adsorption pathway [20,21]. Kinetic approaches, in contrast, can resolve multistep processes and yield mechanistic insights into how proteins interact with nanoparticle surfaces. Recent studies suggest that electrostatic forces frequently dominate initial adsorption, while hydrophobic interactions become increasingly important in stabilizing the final bound state and driving structural rearrangements. This two-step adsorption mechanism has been supported by molecular-level simulations and experiments at solid–water interfaces [22,23,24]. Nevertheless, direct experimental evidence for residue-specific contributions to adsorption-induced unfolding remains limited.

The B1 domain of *streptococcal* protein G (GB1) provides an ideal model for probing such mechanisms. GB1 is small, well-characterized, and extensively studied in protein folding research [25,26,27,28,29], making it an excellent candidate for adapting concepts such as Φ-value analysis to protein–nanoparticle systems. Our previous work examined the global adsorption and unfolding behavior of wild-type GB1 on nanoparticle surfaces, both experimentally [30] and through atomistic simulations [31]. Although these studies focused on global adsorption pathway of wild-type GB1 and identified residue contacts at the interface, they did not provide experimental tests of residue function or quantify how specific mutations alter the energetics and kinetics of adsorption-induced unfolding.

Here, we employed seven site-directed GB1 mutants targeting diverse structural regions—including β-strands, turns, the α-helix, and the C-terminal hairpin—to map the adsorption and unfolding pathway on latex nanoparticles. Using a combination of equilibrium fluorescence, stopped-flow kinetics, and free-energy analysis, we identify the residues that most strongly influence adsorption energetics and unfolding transitions. Our results reveal a residue-specific mechanism in which Asp46 acts as a primary electrostatic docking site, Thr53 modulates the stability of the surface-bound unfolded state, and hydrophobic residues contribute later in the pathway. By integrating kinetic and thermodynamic analysis with Φ-value interpretation, we establish a mechanistic model of protein adsorption-induced unfolding that provides new insights into protein corona formation. Beyond elucidating GB1 behavior, this framework may serve as a general strategy for predicting protein–nanoparticle interactions and guiding the design of nanomaterials with improved biocompatibility.

## 2. Materials and Methods

### 2.1. Chemicals

The sulfate latex particles of 80 nm, supplied as suspension in water at a concentration of 8% *w*/*v*, was purchased from IDC, Invitrogen (Carlsbad, CA, USA). All other chemicals were of reagent grade and directly used in experiments without further purification.

### 2.2. Protein Expression and Purification

The plasmid encoding GB1 mutations genes was kindly provided by Prof. Hongbin Li of the University of British Columbia. GB1 mutations were expressed in BL21 and purified by Ni^2+^-affinity chromatography. The purified protein samples were dialyzed against PBS and kept at 4 °C at concentrations of ~2 mg mL^−1^.

### 2.3. Circular Dichroism (CD)

The CD spectra were obtained on a J-815 spectropolarimeter (JASCO Corporation, Tokyo, Japan), equipped with a Peltier temperature control system set at 25 °C. The secondary structure was followed in the far-UV regime (200–260 nm), using a protein concentration of ~0.1 mg mL^−1^ in water and an optical path of 1 mm. The results were expressed as mean residue molar ellipticity, [θ]. The reported CD spectra were averaged from 3 scans with a response time of 1 s and a spectral bandwidth of 1.0 nm to increase the signal-to-noise ratio.

### 2.4. Fluorescence Spectroscopy

All steady-state emission spectra were obtained with a spectrofluorometer FP-6500 (JASCO Corporation, Tokyo, Japan). Tryptophan emission spectra were excited at 285 nm with emission collected from 300 to 400 nm with a 3 nm bandwidth. The background fluorescence from latex nanoparticles has been subtracted in the reported spectra. Equilibrium binding isotherms were determined from at least three independent titrations for each mutant using a protein concentration of 0.03 mg/mL.

### 2.5. Stopped-Flow Measurement

The kinetic measurements were carried out by using a stopped-flow apparatus with a MOS-450 light-chamber and an SFM-300 device (Bio-Logic, Seyssinet-Pariset, France) as described previously. The stopped-flow apparatus was equipped with three motor-controlled syringes filled with 0.1 mg/mL protein solution, PBS buffer, and 0.075% *w*/*v* latex nanoparticle solution, respectively. In a typical experiment, desired amount of protein solution, buffer and nanoparticle solution was pushed by the syringes through a high-density solution mixer (HDS) to a quartz sample chamber. The typical mixing dead time was ~3 ms. The mixed solution was stopped in the sample chamber to observe the structural change in proteins upon mixing with latex nanoparticles by monitoring its intrinsic fluorescence excited at 285 nm with a 320 nm cut-off emission filter. The measurements were duplicated at least three times for each mutant and every kinetic profile was obtained from an average of four to six stopped-flow shots. All averaged traces were fitted with a double-exponential equation to extract observed rate constants k_1obs_ and k_2obs_.

### 2.6. Obtaining Apparent Dissociation Constant from Equilibrium Binding Isotherm

The binding isotherm of GB1 on latex nanoparticle surface was fitted with the following equation:

(1) $$Bound\%=\frac{[GB1]+n[latex]+K_{ed}-\sqrt{{\left([GB1]+n[latex]+K_{ed}\right)}^{2}-4[GB1]\times n[latex]}}{2[GB1]}$$

where Bound% is the fraction of bound GB1 in the solution; [GB1] is the initial GB1 concentration in the unit of M; [latex] is initial concentration of latex nanoparticle in the concentration of (*w*/*v*); n is a prefactor, which converts the concentration of latex nanoparticles to the concentration of total available binding sites on latex nanoparticles in solution; and K_ed_ is the apparent dissociation constant.

### 2.7. Obtaining Kinetic Parameters Using Two-Step Revisable Model

According to the model, we are able to quantitatively obtain adsorption rate, k_on_, desorption rate, k_off_, folding rate, k_f_, and unfolding rate, k_u_, from the observed rate constant k_1obs_ and k_2obs_ as follows:

(2) $$k_{1obs}=k_{on}\times (n[Latex]+[GB1])+k_{off}$$

(3) $$k_{2obs}=k_{f}+\frac{k_{u}\times (n[Latex]+[GB1])}{k_{off}/k_{on}+n[Latex]+[GB1]}$$

where k_1obs_, k_2obs_, k_on_, k_off_, k_f_, and k_u_ are defined above; [latex] and [GB1] represents the concentration of latex nanoparticles with a unit of *w*/*v* and the concentration of GB1 with a unit of M, respectively.

## 3. Results and Discussions

### 3.1. Native Fold Preservation and Surface-Induced Unfolding of GB1 Variants

**Figure 1:**
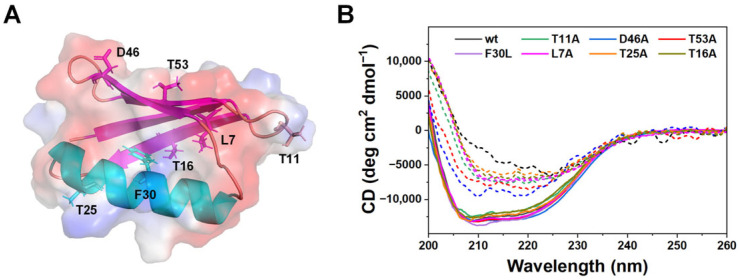
Structural mapping and secondary structure characterization of GB1 variants. (**A**) Structural model of GB1 showing the locations of single-site mutations used in this study. The β-sheet is shown in magenta and the α-helix in cyan, with the protein surface shaded to emphasize electrostatic features. (**B**) Far-UV CD spectra of wild-type GB1 (black) and all single-site mutants, measured before (solid lines) and after adsorption onto 0.005% *w*/*v* latex nanoparticles (dashed lines). The data of wild-type GB1 was adapted from ref. [30].

To establish that mutagenesis did not disrupt the native fold of GB1, we mapped the positions of the engineered residues onto the GB1 structure (Figure 1A). The substitutions span three functional categories: electrostatic residues (Asp46, Thr53), hydrophobic-core residues (Phe30, Thr25), and distal positions (Leu7, Thr11, Thr16). This distribution ensured that both putative adsorption hot spots and control positions were probed.

Far-UV circular dichroism (CD) spectra confirmed that all seven variants preserved the characteristic β-grasp fold in solution, with spectral minima near 210–220 nm indistinguishable from the wild type (Figure 1B, solid lines). Thus, the introduced mutations do not compromise the global stability or secondary structure of GB1 in the ground state. Upon adsorption onto latex nanoparticles, all variants exhibited marked loss of secondary structure (Figure 1B, dashed lines), demonstrating that unfolding is surface-induced rather than due to intrinsic instability of the mutants [30,32,33,34,35].

These results align with prior observations that most single-site substitutions in GB1 leave the global fold intact while tuning stability or kinetic behavior [26,36]. Preserving structural integrity is critical for mechanistic interpretation: since the variants are well folded in solution, the unfolding and kinetic differences observed in later experiments can be attributed directly to altered nanoparticle interactions rather than pre-existing destabilization, aggregation or quenching effects. This interpretation is further supported by prior studies across a range of nanoparticle systems, which demonstrate that CD signal loss upon protein-nanoparticle binding reliably reflects genuine secondary structure disruption rather than nonspecific light scattering or fluorescence interference [37,38,39].

### 3.2. Adsorption Equilibria and Binding Affinity of GB1 Variants

**Figure 2:**
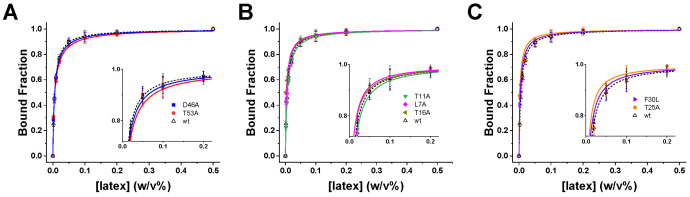
Adsorption equilibria of GB1 variants on latex nanoparticles. Equilibrium binding isotherms measured by fluorescence titration for wild-type GB1 (black) and single-site variants. Data are grouped by functional category: (**A**) electrostatic residues (D46A, T53A), (**B**) distal mutations (T11A, L7A, T16A), and (**C**) hydrophobic-core residues (F30L, T25A). Insets show magnified views of the transition regions, highlighting differences relative to wild type. Error bars represent standard deviations from 3 replicate measurements; solid lines correspond to fitted binding isotherms used to extract the apparent dissociation constants (K_ed_ (M)) using Equation (1). The wild-type GB1 data was adapted from [30] showed in dashed lines.

We next assessed how individual mutations modulate the adsorption equilibrium of GB1 onto latex nanoparticles. Fluorescence titrations were performed to quantify binding isotherms for each variant (Figure 2), with dissociation constants (K_ed_) determined from global fits by Equation (1) (Section 2). To highlight functional contributions, the data are grouped into three categories: electrostatic residues, distal mutations, and hydrophobic-core.

The equilibrium binding curves satisfy the criteria for the titration regime [40], with bound fractions spanning the full dynamic range and low residuals across two orders of magnitude in latex concentration, supporting the reliability of the extracted K_ed_ values. Electrostatic mutants (D46A, T53A) exhibited the most pronounced changes, with isotherms shifted to higher nanoparticle concentrations relative to wild type (Figure 2A). The corresponding K_ed_ values increased by ~1.2–1.5-fold (Table 1), confirming that electrostatic interactions at Asp46 and Thr53 play a central role in initiating adsorption. By contrast, distal mutations (L7A, T11A, T16A) produced isotherms nearly overlapping with wild type (Figure 2B). The insets reveal only minor deviations, indicating negligible contributions to equilibrium binding. Hydrophobic-core mutations (F30L, T25A) produced modest effects (Figure 2C). While the isotherms largely resembled wild type, subtle rightward shifts in the insets suggest a small but detectable weakening of affinity, consistent with their role in stabilizing later stages of the adsorption process rather than initial docking.

Taken together, the equilibrium data demonstrate that adsorption is initiated primarily by electrostatic recognition at the hairpin residues, with hydrophobic contacts playing a subsidiary role in stabilizing the adsorbed ensemble and distal positions having minimal influence on binding thermodynamics [20,22,41].

### 3.3. Kinetic Pathways of Adsorption-Induced Unfolding

While equilibrium isotherms capture the overall binding affinity and residue-specific thermodynamic contributions, they provide limited insight into the temporal sequence of adsorption and conformational rearrangements. Adsorption to nanoparticle surfaces is inherently a dynamic process, in which recognition, attachment, and structural reorganization may occur on distinct timescales. To establish the mechanistic framework for interpreting these dynamics, we summarize the reversible two-step pathway governing the process:

$$N\overset{k_{on}}{\underset{k_{off}}{⇄}}N_{s}\overset{k_{u}}{\underset{k_{f}}{⇄}}U_{s}$$

where N denotes the native protein in solution, N_s_ the native-like adsorbed intermediate, and U_s_ the unfolded surface-bound ensemble.

**Figure 3:**
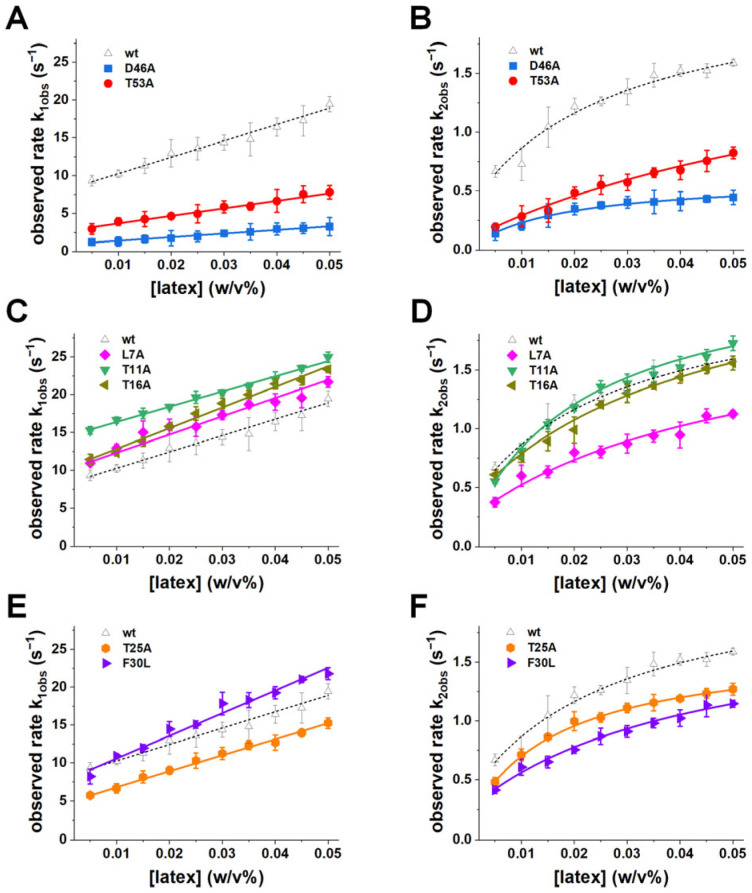
Kinetic rates of adsorption-induced unfolding. Observed rates of the fast (k_1obs_ (s^−1^), left panels) and slow (k_2obs_ (s^−1^), right panels) kinetic phases are plotted as a function of latex nanoparticle concentration for wild type and GB1 variants. (**A**,**B**) Electrostatic mutants D46A (blue) and T53A (red). (**C**,**D**) Distal mutants L7A (magenta), T11A (green), and T16A (brown). (**E**,**F**) Hydrophobic-core mutants T25A (orange) and F30L (purple). Wild-type data shown in dashed black lines was adapted from ref. [30]. Solid lines indicate fits to the kinetic model (Equations (2) and (3)) to extract adsorption (k_on_ (M^−1^·s^−1^)), desorption (k_off_ (s^−1^)), unfolding (k_u_ (s^−1^)), and refolding (k_f_ (s^−1^)) rate constants (Table 2). Error bars represent standard deviations from at least three independent measurements.

To resolve these kinetic steps and directly visualize how mutations alter the adsorption pathway, we next employed time-resolved fluorescence measurements using a stopped-flow approach. Stopped-flow fluorescence kinetics revealed biphasic behavior across all GB1 variants, with a fast adsorption phase followed by a slower unfolding phase (Figure S1). These kinetic phases were globally fitted to the two-step reversible model (Equations (2) and (3) in Section 2) to obtain the microscopic rate constants for adsorption (k_on_), desorption (k_off_), unfolding on the surface (k_u_), and refolding on the surface (k_f_) (Table 2). Proteins exhibited rapid adsorption (high k_on_) followed by a slower unfolding transition (k_u_), consistent with expectation for proteins interacting with charged nanoparticle surfaces (Figure 3A–F) [42,43]. Both kinetic steps are reversible, as evidenced by measurable k_off_ values for desorption and nonzero k_f_ values indicating refolding from the surface-induced unfolded state. These observations are consistent with prior experimental demonstrations of reversible adsorption-induced unfolding [30].

Electrostatic mutations caused the most pronounced kinetic perturbations. D46A displayed a dramatic ~6-fold reduction in k_on_ (from 17.87 ± 1.14 to 2.87 ± 0.18 M^−1^ s^−1^) and an approximately ~10-fold reduction in k_off_ (from 8.20 ± 0.42 to 0.89 ± 0.09 s^−1^) (Figure 3A, Table 2). These changes reflect a profound impairment in both productive docking and desorption, indicating that loss of Asp46’s electrostatic contribution raises the free-energy barrier for the adsorption transition and slows the resolution of the nanoparticle-bound state. T53A also slowed both adsorption and unfolding kinetics, though to a lesser extent, with a ~3-fold reduction in k_on_ and a ~4-fold reduction in k_off_ (Figure 3A, Table 2). These kinetic perturbations confirm that Asp46 and Thr53 are critical hotspots for controlling the adsorption pathway. The loss of electrostatic stabilization in D46A not only weakens the initial docking but also slows subsequent structural transitions, leading to a significantly reduced rate of nanoparticle-bound state resolution. T53A, by destabilizing the β-hairpin, likely hinders the conformational flexibility required for efficient unfolding after binding.

In contrast, distal mutants (L7A, T11A, T16A) exhibited k_on_ and k_off_ values nearly identical to wild type, with minimal deviations in the kinetic traces (Figure 3C, Table 2). This indicates that these residues do not significantly impact either the adsorption or unfolding steps, emphasizing their negligible role in nanoparticle interactions.

Hydrophobic-core mutations (T25A, F30L) led to modest increases in k_on_ and k_u_ (Figure 3E, Table 2), suggesting that these residues contribute primarily to the later stabilization of the surface-bound state rather than to the initial electrostatic docking. F30L, in particular, showed slightly higher k_on_ and k_u_ compared to wild type, consistent with enhanced hydrophobic interactions upon surface exposure.

Beyond the initial adsorption step, mutations also reshaped the unfolding–refolding equilibrium on the nanoparticle surface. Comparing k_u_ and k_f_ values revealed that wild-type GB1 favors the unfolded-on-surface ensemble, with a k_u_/k_f_ ratio of ~5.9. D46A decreased both k_u_ and k_f_ but suppressed refolding disproportionately, producing k_u_/k_f_ ≈ 50.8 and thereby effectively trapping the protein in the unfolded ensemble (Figure 3B, Table 2). T53A similarly increased the unfolded state preference (k_u_/k_f_ ≈ 23.6, ~4× wild type), which is consistent with destabilization of the β-hairpin and impaired refolding (Figure 3B, Table 2). Hydrophobic and distal mutants produced smaller shifts in k_u_/k_f_ (typically ~6–10, less than 2-fold of wild type), indicating subtle stabilization of the unfolded state without strongly perturbing the adsorption step (Figure 3D,F, Table 2).

These observations highlight that electrostatic and hairpin mutations not only slow adsorption but also destabilize the refolding branch of the pathway, strongly biasing the equilibrium toward the unfolded-on-surface state. In contrast, hydrophobic mutations subtly favor unfolding without significantly affecting the adsorption barrier. Thus, adsorption reshapes the energy landscape such that specific mutations can determine whether the surface-bound protein remains flexible or becomes kinetically trapped in an unfolded ensemble. The biphasic kinetic behavior, consisting of a rapid adsorption phase followed by slower unfolding, is consistent with what has been observed in other protein–nanoparticle systems [44]. Our analysis of k_on_, k_off_, k_u_, and k_f_ provides direct, residue-specific mechanistic evidence for how different structural regions of GB1 contribute to each step of the adsorption-induced unfolding pathway. The existence of a metastable intermediate (partially folded on the nanoparticle surface) and the staged nature of the unfolding pathway emphasize how nanoparticles can act as catalysts for protein misfolding, where adsorption serves as the initial step in driving the protein toward a misfolded state.

### 3.4. Φ-Value Analysis of Adsorption Transition States

To localize residue-level energetic contributions along the adsorption–unfolding pathway, we performed a Φ-value analysis using kinetic and equilibrium parameters derived from the two-step model. Here, the factor Φ is defined as Φ_i_ = ΔΔG_i_/ΔΔG_f_ − 1, where ΔΔG_i_ represents the free energy change in the mutant relative to wild type at the transition or state of interest, and ΔΔG_f_ is the folding free energy difference between wild type and mutants (Figure S2, Tables S1 and S2). This definition differs from classical φ-value analysis in two-state folding [45,46,47] by accounting for adsorption-induced energetic changes separately from intrinsic folding stability. It reflects the fraction of the mutation’s energetic effect expressed at a specific transition or state: a positive Φ indicates strong energetic involvement, a Φ near zero suggests minimal contribution, and a negative Φ implies an opposite or compensatory effect relative to the folded protein in solution. Applying this framework to the adsorption–unfolding pathway, we determined four Φ values corresponding to key energetic stages: Φ_ads_ for N → T_ads_ transition, Φ_Ns_ for the adsorbed intermediate N_s_, Φ_ads-U_ for N_s_ → T_ads-U_ transition, and Φ_Us_ for the unfolded-on-surface ensemble U_s_ (Table 3). Here, T_ads_ and T_ads–U_ refer to the transition states associated with initial adsorption and surface-induced unfolding, respectively, as defined in the reversible two-step kinetic pathway (Figure S2).

Across the N → T_ads_ transition, D46A exhibited a strongly positive Φ_ads_ (0.63), underscoring its dominant role in electrostatic anchoring during early docking. Although both Asp46 and the sulfate-modified nanoparticle surface are negativelycharged, adsorption likely proceeds through cation-mediated bridging or short-range interactions (e.g., van der Waals interactions [31]) rather than direct Coulomb attraction, explaining the pronounced kinetic perturbation upon mutation. Consistent with this mechanism, our previous work demonstrated that increasing the solution pH from 7 to 9 reduces GB1 adsorption efficiency—reflecting enhanced protein net negative charge and weakened electrostatic engagement—while elevating NaCl concentration progressively decreases the apparent adsorption rate constant (k_on_) indicating electrostatic screening and disruption of cation-mediated bridging [30]; together, these effects quantitatively support the role of electrostatic interactions in governing the N → T_ads_ transition. T53A displayed a modest positive Φ_ads_ (0.26), suggesting a contributing role in stabilizing the transition state at the C-terminal hairpin, whereas hydrophobic and distal mutations (F30L, T25A, T11A, T16A, L7A) yielded small or negative values (−0.01 to −1.05), indicating minimal involvement at this stage. These results establish electrostatic interactions—dominated by Asp46 and supported by Thr53—as the primary determinants of the early adsorption.

The Φ_Ns_ values reveal an energetic redistribution after docking. T25A (0.32) and T11A (0.53) showed positive contributions, indicating their involvement in stabilizing the adsorbed intermediate. In contrast, D46A (−0.14), T53A (−0.09), and F30L (−0.26) showed negative values, suggesting diminished roles of electrostatic and hydrophobic core residues once the protein is surface-attached. L7A and T16A contributed minimally. These trends point to a shift from electrostatic control to loop-mediated stabilization within the surface-bound intermediate.

For the N_s_ → T_ads-U_ transition, Φ_ads-U_ values reveal a gradual shift in energetic contributions. D46A (0.30) remained the dominant contributor, reflecting persistent electrostatic effects beyond initial docking. T53A (0.14) contributed modestly, indicating that the C-terminal hairpin continues to influence the unfolding barrier. T11A (0.08) showed a weak but sustained contribution consistent with its role in stabilizing N_s_, whereas T16A (0.08) likely reflects a minor, nonspecific effect. T25A (−0.64) exhibited a negative contribution, suggesting structural rearrangement upon unfolding, and F30L (−0.08) contributed minimally. These results indicate a progressive handover from electrostatic anchoring to loop-mediated interactions during the unfolding transition.

The Φ_Us_ values show the broadest distribution, reflecting the heterogeneity of the unfolded-on-surface ensemble. T25A displayed a strongly positive value (0.50), marking it as a dominant contributor to U_s_ stabilization. D46A (−0.88), T53A (−0.53), and F30L (−0.44) yielded negative values, indicating their stabilizing roles in the wild-type unfolded ensemble are lost upon mutation. L7A, T11A, and T16A exhibited smaller effects, suggesting modest contributions. This pattern illustrates how hydrophobic and electrostatic residues stabilize the final ensemble through different mechanisms.

**Figure 4:**
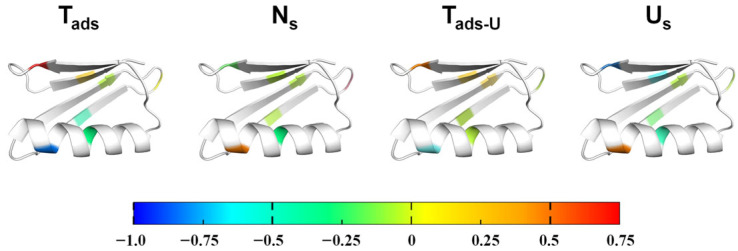
Residue-level Φ-value mapping during adsorption-induced unfolding. Φ-values were mapped onto the GB1 structure for T_ads_, N_s_, T_ads-U_, and U_s_ states. The color bar (−1.0 to +0.75) indicates energetic contributions: positive (red–orange) reflects strong involvement of residues at a given state, near-zero (green) indicates minimal contribution, and negative (blue) reflects compensatory or opposite energetic effects due to surface interactions. This visualization highlights how different residues contribute across successive stages of adsorption-induced unfolding.

Mapping these Φ-values onto the GB1 structure (Figure 4) reveals a clear spatial and temporal polarization of residue contributions along the adsorption pathway. Electrostatic residues in the C-terminal β-hairpin, particularly Asp46 and Thr53, dominate the early N → T_ads_ transition, establishing the initial adsorption interface. In contrast, residues Thr11 and Thr25, located near the loop and β-sheet interface, contribute most prominently to stabilizing the adsorbed intermediate and the N_s_ → T_ads-U_ transition. Hydrophobic core residues such as Phe30 exert their influence predominantly at the unfolded-on-surface state, indicating their role in stabilizing the final ensemble rather than shaping kinetic barriers. This redistribution of energetic contributions delineates a two-step mechanism in which electrostatic anchoring initiates adsorption, followed by hydrophobic-assisted unfolding and stabilization on the nanoparticle surface. The alignment of these energetic “hot spots” with regions previously identified as part of the GB1 folding nucleus further suggests that nanoparticle adsorption redirects the native folding energy landscape toward a surface-mediated unfolding pathway, rather than creating a fundamentally distinct process.

Comparison of our Φ-value analysis with established GB1 folding studies further highlights how nanoparticle adsorption reshapes—but does not entirely replace—the energetic motifs that govern folding in solution. McCallister et al. [26] demonstrated that the second β-hairpin (residues 41–56), particularly positions such as Asp46 and Thr49/53, forms the core of the folding nucleus, exhibiting high Φ-values and dominating the N → T_s_ transition during solution folding. Consistent with this intrinsic hierarchy, our adsorption Φ_ads_ values identify Asp46 and Thr53 as the major contributors to the early N → T_ads_ transition, indicating that the C-terminal hairpin also nucleates the initial docking interaction with the nanoparticle surface. However, as the protein progresses from T_ads_ → N_s_ → T_ads–U_ → U_s_, the energetic weight shifts toward loop and hydrophobic residues (e.g., Thr25, Thr11, Phe30), which make minimal contributions to the solution folding transition state but become increasingly important for stabilizing surface-bound intermediates and the unfolded-on-surface ensemble. This comparison suggests that nanoparticle adsorption preserves the energetic prominence of the native folding nucleus during the initial docking step while redirecting the subsequent energetic landscape to incorporate residues that specifically stabilize interfacial unfolding states.

### 3.5. Mechanistic Model of GB1 Adsorption and Unfolding

**Figure 5:**
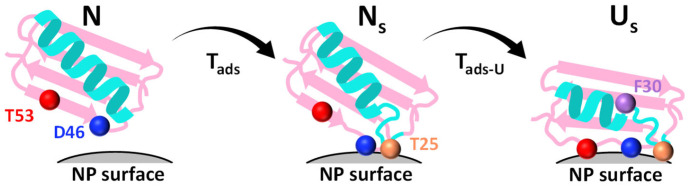
Mechanistic model of GB1 adsorption and unfolding. Schematic of the dock–loosen–unfold pathway derived from Φ-value analysis. GB1 transitions from the native state (N) to a native-like adsorbed intermediate. (N_s_) via transition T_ads_, and then to the unfolded-on-surface state (U_s_) via transition T_ads–U_. Residues Asp46 (blue) and Thr53 (red) dominate the initial docking, loop residue Thr25 (orange) stabilizes the intermediate, and hydrophobic Phe30 (purple) consolidates the unfolded ensemble on the nanoparticle surface.

Integrating the equilibrium, kinetic, and Φ-value analyses yield a coherent mechanistic model of GB1 adsorption and unfolding on nanoparticle surfaces (Figure 5). The pathway begins with electrostatic docking, dominated by Asp46, which acts as a gatekeeper residue that lowers the barrier for productive nanoparticle engagement. This early stage already involves partial structuring of the C-terminal hairpin (Thr53), as revealed by positive Φ-values, indicating that local hairpin geometry is central in the adsorption transition state. Together, these residues define an adsorption nucleus that channels the protein onto the nanoparticle surface.

Following this initial recognition, the protein enters a metastable intermediate (N_s_) in which it is fully surface-attached but only partially unfolded. In this state, electrostatic contacts anchor the protein, while gradual loosening of the hydrophobic core begins to expose residues such as Phe30 and Thr25. These hydrophobic contacts progressively stabilize the surface-associated ensemble, bridging the transition from docking to unfolding.

Ultimately, the protein transitions to the unfolded-on-surface state (U_s_), in which large segments are disordered yet tightly bound. Here, hydrophobic residues play a decisive role in stabilizing the final corona-associated ensemble, while Thr53 helps maintain the geometry of the unfolded hairpin against the surface, shaping the kinetic partitioning between unfolding and refolding. The k_u_/k_f_ analyses underscore that mutations at D46 and T53 bias the energy landscape to favor persistent unfolding, highlighting their disproportionate influence on both the transition state and the final bound ensemble.

This integrated model can be summarized as a dock–loosen–unfold sequence: electrostatic hairpin residues initiate surface recognition, hydrophobic core exposure consolidates adsorption, and C-terminal hairpin stabilization locks the unfolded state onto the nanoparticle. The parallels with folding nuclei in solution are striking—just as pre-organized local structures govern folding pathways, pre-formed hairpin elements guide adsorption and unfolding on surfaces. Such residue-specific insights bridge folding theory with interfacial biophysics, offering a unified framework for interpreting how proteins misfold or reorganize at interfaces.

This mechanism also aligns with broader findings from recent experimental and computational studies of protein–nanoparticle interactions. Cryo-electron microscopy and tomography have revealed that the biomolecular corona exhibits structural heterogeneity and dynamic conformational rearrangements at the nanoscale, highlighting how adsorption can alter local protein architecture depending on surface properties and binding geometry [48]. Molecular simulations further demonstrate that proteins adopt distinct adsorption strategies—some relying on flexible loops and hydrophilic patches, others engaging via stable hydrophobic cores—depending on surface chemistry and topology [49]. These observations support a generalized model in which electrostatics guide initial recognition, while hydrophobic contacts consolidate binding and induce structural reorganization. Our Φ-value results for GB1 adsorption mirror this progression and illustrate how individual residues can disproportionately influence each stage, depending on their local environment and structural role.

While our two-step kinetic model assumes sequential adsorption and unfolding, it is possible that these events exhibit some temporal overlap under certain conditions. However, the presence of distinct fast and slow kinetic phases in our fluorescence data—with different nanoparticle concentration dependencies—supports their kinetic separability. Furthermore, because the Φ-values reflect comparative energetic shifts between wild-type and mutant pathways, they remain informative even if some structural transitions are partially coupled. Nevertheless, we acknowledge that future studies using time-resolved structural probes or simulations could further refine the transition sequence and resolve any microscopic overlap between adsorption and unfolding steps.

Surface charge or material composition of the nanoparticle (e.g., silica, gold, or cationic latex) could modulate the dock–loosen–unfold mechanism by altering which interactions dominate each stage of adsorption. For instance, a positively charged interface (such as a cationic latex nanoparticle) might engage a different set of protein residues during the initial electrostatic docking compared to those observed on the negatively charged sulfate latex surface used here, while hydrophilic silica or inert gold nanoparticles could shift the balance between electrostatic anchoring and hydrophobic stabilization in later unfolding steps. Although our current study focuses on negatively charged sulfate latex nanoparticles, the core residue-resolved framework remains broadly applicable across different nanoparticle interfaces, , and systematic comparison across materials represents an important direction for future work. From a design perspective, tailoring nanoparticle surface charge could suppress electrostatic docking, reduce corona-driven unfolding, and improve nanomaterial biocompatibility [50]. Conversely, deliberate modulation of hydrophobicity or hairpin stabilization could be exploited in biosensing applications that rely on controlled protein destabilization [8,51]. In addition to their mechanistic implications, these insights provide a predictive basis for controlling corona formation through rational tuning of surface charge and hydropho-bicity to minimize protein destabilization in therapeutic nanoparticles, reducing immu-nogenicity and improving safety [44], while adsorption-induced unfolding pathways may also be harnessed for functional signal amplification in nanoparticle-based sensing systems [52]. In either context, the combined use of mutational analysis, kinetics, and Φ-value mapping provides a generalizable strategy to dissect and engineer protein–nanoparticle interactions.

## 4. Conclusions

This study provides a residue-specific sampling of key sites that shape the kinetic and energetic pathway by which GB1 unfolds upon adsorption to nanoparticle surfaces. By integrating equilibrium binding, stopped-flow kinetics, and Φ-value analysis, we reveal a dock–loosen–unfold mechanism governed by distinct residue classes. Electrostatic anchoring at Asp46 initiates surface recognition, Thr53 in the C-terminal hairpin mediates structural loosening, and hydrophobic residues such as Phe30 and Thr25 stabilize the unfolded-on-surface ensemble. These results map the temporal and energetic sequence of adsorption-induced unfolding with unprecedented detail, showing that nanoparticle binding effectively redirects the native folding energy landscape toward a surface-driven pathway. More broadly, the combined kinetic, thermodynamic, and mutational framework established here offers a general approach for dissecting adsorption-induced conformational transitions at bio–nano interfaces.

## Figures and Tables

**Table 1:** Thermodynamic binding parameters for GB1 variants interacting with latex nanoparticle surfaces in PBS buffer, determined from equilibrium adsorption isotherms.

	wt *	D46A	T53A	F30L	T25A	L7A	T11A	T16A
K_ed_ (M)	0.069 ± 0.007	0.101 ± 0.012	0.085 ± 0.004	0.048 ± 0.002	0.066 ± 0.003	0.058 ± 0.003	0.076 ± 0.004	0.074 ± 0.002
n (M (*w*/*v*)^−1^)	11.7 ± 1.2	16.82 ± 2.3	13.56 ± 1.6	9.56 ± 1.4	17.01 ± 2.6	11.59 ± 1.2	11.2 ± 1.1	12.64 ± 1.3

* Data of wt GB1 was adapted from ref. [30].

**Table 2:** Summary of the kinetic parameters for the adsorption of GB1 variants on latex nanoparticle surfaces in PBS buffer.

	wt *	D46A	T53A	F30L	T25A	L7A	T11A	T16A
k_off_ (s^−1^)	8.20 ± 0.42	0.89 ± 0.09	2.61 ± 0.14	7.76 ± 0.48	4.95 ± 0.23	10.90 ± 0.45	14.37 ± 0.21	10.11 ± 0.63
k_on_ (M^−1^·s^−1^)	17.87 ± 1.14	2.87 ± 0.18	7.65 ± 0.32	30.91 ± 1.60	12.02 ± 0.44	18.13 ± 1.25	18.02 ± 0.61	21.69 ± 1.55
k_f_ (s^−1^)	0.38 ± 0.08	0.012 ± 0.04	0.044 ± 0.011	0.16 ± 0.03	0.25 ± 0.05	0.19 ± 0.04	0.34 ± 0.03	0.33 ± 0.04
k_u_ (s^−1^)	2.24 ± 0.18	0.61 ± 0.09	1.04 ± 0.09	1.45 ± 0.07	1.56 ± 0.08	1.93 ± 0.11	3.52 ± 0.12	2.10 ± 0.11

* Data of wt GB1 was adapted from ref. [30].

**Table 3:** Φ-values for GB1 variants at four stages of the adsorption pathway: Φ_ads_ (N → T_ads_), Φ_Ns_ (N_s_), Φ_ads-U_ (N_s_ → T_ads-U_), and Φ_Us_ (U_s_), reflecting residue-specific energetic contributions.

Φ	wt	D46A	T53A	F30L	T25A	L7A	T11A	T16A
Φ_ads_	-	0.63	0.26	−0.23	−1.05	−0.01	−0.02	−0.42
Φ_Ns_	-	−0.14	−0.09	−0.26	0.32	0.09	0.53	0
Φ_ads-U_	-	0.3	0.14	−0.08	−0.64	0.13	0.08	0.08
Φ_Us_	-	−0.88	−0.53	−0.44	0.5	−0.1	−0.03	−0.16

## Data Availability

The original contributions presented in this study are included in the article/Supplementary Material. Further inquiries can be directed to the corresponding authors.

## Supplementary Materials

The following supporting information can be downloaded at: https://www.mdpi.com/article/10.3390/biom16010114/s1, Figure S1: Typical normalized stopped-flow traces of GB1 variants; Figure S2: Energy landscape for ΔG and ΔΔG analysis; Table S1: Summary of ∆G values for GB1 variants at four stages of the adsorption pathway; Table S2: Summary of ∆∆G values for GB1 variants at four stages of the adsorption pathway.

## Abbreviations

The following abbreviations are used in this manuscript:

GB1	The B1 domain of streptococcal protein G
Far-UV	Far ultraviolet
CD	Circular dichroism
