# Supplementary material for: Residue-Specific Dock-Loosen-Unfold Mechanism of GB1 on Nanoparticle Surfaces Revealed by Kinetic and Φ-Value Analysis
Source: Biomolecules. 2026 Jan 8;16(1):114. doi: 10.3390/biom16010114 (PMC12839133; doi:10.3390/biom16010114)
Supplement: Supplementary file 1 [file biomolecules-16-00114-s001.zip › biomolecules-3989774-supplementary.pdf]

Supporting Information

Residue-Specific Dock-Loosen-Unfold Mechanism of GB1 on Nanoparticle Surfaces Revealed by Kinetic and Φ-Value Analysis

Tingting Liu[1, 2], Yunqiang Bian[1], Siyu Wang[1, 2], Yang Li[1], Yi Cao[1, 3], Yonghua Jiao[2, *], Hai Pan[1, *]

[1] Wenzhou Institute, University of Chinese Academy of Sciences, Wenzhou, Zhejiang 325001, China
[2] College of Life and Health Sciences, Northeastern University, Shenyang, Liaoning 110819, China
[3] Chemistry and Biomedicine Innovation Center, Nanjing University, Nanjing, Jiangsu 210093, China

[*] Corresponding author. Email: hpan@ucas.ac.cn; or jiaoyh@mail.neu.edu.cn

ADDITIONAL METHOD

Free Energy Calculation of ΔG_i and $\Delta\Delta G_i$: Free energy changes for each step of the adsorption–unfolding pathway were derived from kinetic and equilibrium parameters obtained from the two-step model (Figure S2). For the initial adsorption step (N → N_s), the standard Gibbs free energy ($\Delta G_{N \rightarrow N_s}$) was calculated from the equilibrium constant $K_1 = \frac{k_{\text{on}}}{k_{\text{off}}}$ as:

$$\Delta G_{N \rightarrow N_s} = -RT\ln K_1 = -RT\ln\left(\frac{k_{\text{on}}}{k_{\text{off}}}\right)$$

where R is the gas constant (1.9872×10^{-3} kcal·mol^{-1}·K^{-1}) and T is the absolute temperature (300 K in our case), and RT is taken as 0.5962 kcal·mol^{-1}.

For the unfolding step (N_s → U_s), the free energy change ($\Delta G_{N_s \rightarrow U_s}$) was calculated similarly using $K_2 = \frac{k_u}{k_f}$:

$$\Delta G_{N_s \rightarrow U_s} = -RT\ln K_2 = -RT\ln\left(\frac{k_u}{k_f}\right)$$

The mutation-induced free energy differences ($\Delta\Delta G_i$) at each transition or state $i$were determined as:

$$\Delta\Delta G_i = \Delta G_i^{\text{mut}} - \Delta G_i^{\text{WT}}$$

where i corresponds to the four key energetic positions: T_{ads} (adsorption transition state), N_s (adsorbed native-like intermediate), $T_{ads\text{–}U}$ (unfolding transition state), and U_s (unfolded-on-surface ensemble). All calculated ΔG_i and $\Delta\Delta G_i$ values for wild type and mutants are summarized in Table S1 and Table S2, respectively.

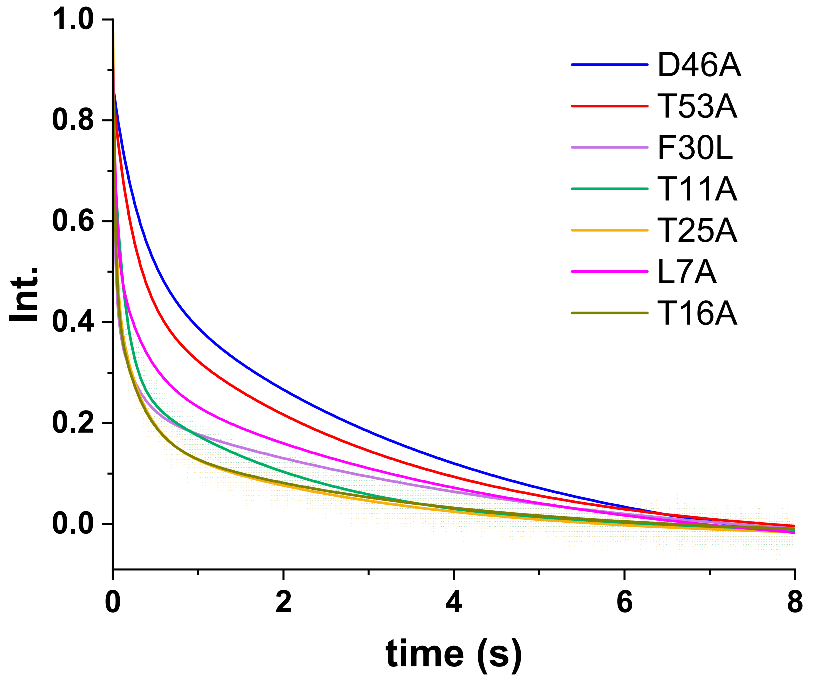

Figure S1. Typical normalized stopped-flow traces of GB1 variants adsorption on latex nanoparticles of 0.05% w/v concentrations in a PBS buffer at pH 7.4. Each trace shown is the average of four to six independent measurements and is fitted with a double exponential function (solid line).

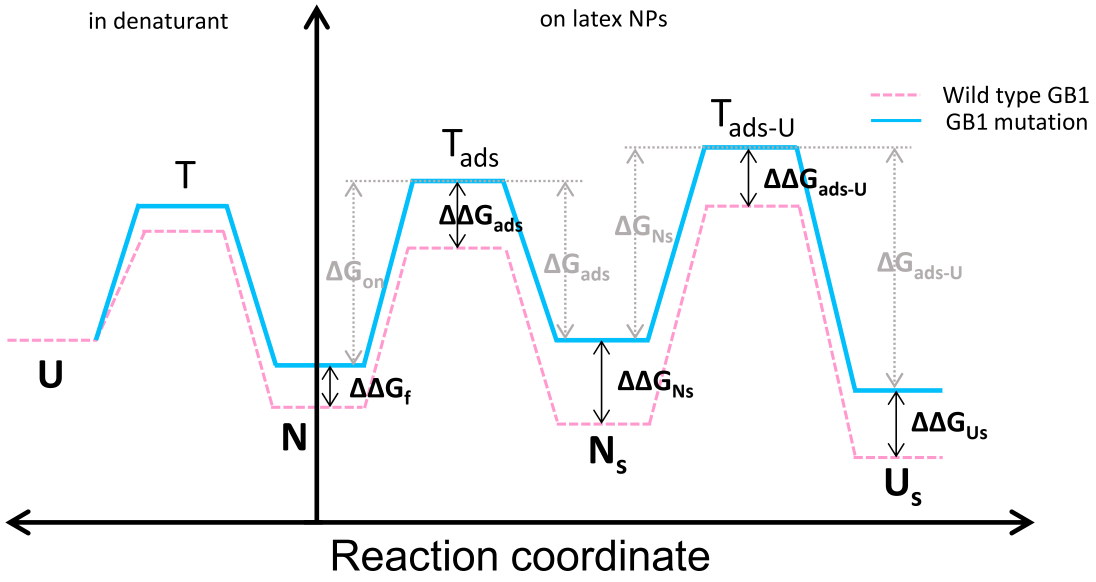

Figure S2. Energy landscape for ΔG and ΔΔG analysis. Schematic representation of the adsorption–unfolding pathway for wild-type GB1 (dashed line) and variants (solid line). The process proceeds from N → T_{ads} → N_s → $T_{ads\text{-}U}$ → U_s. Dashed gray arrows indicate ΔG values for each step derived from kinetic and equilibrium parameters for GB1 wild-type or variants, while black arrows indicate ΔΔG obtained by subtracting mutant energies from wild type. The energy of native state was set to zero to simplify the calculations.

Table S1. Summary of ΔG values for GB1 variants at four stages of the adsorption pathway.

ΔG (kcal·mol^{-1})	**wt**	**D46A**	**T53A**	**F30L**	**T25A**	**L7A**	**T11A**	**T16A**
ΔG_{on}	6.52	7.61	7.02	6.19	6.75	6.51	6.51	6.4
ΔG_{ads}	6.98	8.31	7.66	7.02	7.28	6.81	6.62	6.86
ΔG_{Ns}	7.76	8.53	8.21	8.02	7.97	7.84	7.49	7.79
$\Delta G_{ads\text{-}U}$	8.81	10.87	10.1	9.33	9.06	9.23	8.88	8.9

Table S2. Summary of ΔΔG values for GB1 variants at four stages of the adsorption pathway.

ΔΔG (kcal·mol^{-1})	**wt**	**D46A**	**T53A**	**F30L**	**T25A**	**L7A**	**T11A**	**T16A**
*$\Delta\Delta G_{f}$	-	1.74	1.91	1.42	-0.22	1.85	0.6	0.38
$\Delta\Delta G_{ads}$	-	2.83	2.41	1.09	0.01	1.84	0.59	0.22
$\Delta\Delta G_{Ns}$	-	1.5	1.73	1.05	-0.29	2.01	0.92	0.38
$\Delta\Delta G_{ads\text{-}U}$	-	2.27	2.18	1.31	-0.08	2.09	0.65	0.41
$\Delta\Delta G_{Us}$	-	0.21	0.89	0.79	-0.33	1.67	0.58	0.32

* These values were adapted from reference [26].
